# Intestinal microbiota regulates the gut-thyroid axis: the new dawn of improving Hashimoto thyroiditis

**DOI:** 10.1007/s10238-024-01304-4

**Published:** 2024-02-22

**Authors:** Xiaxin Zhu, Chi Zhang, Shuyan Feng, Ruonan He, Shuo Zhang

**Affiliations:** 1 Zhejiang Chinese Medical University, Hangzhou, 310053 People’s Republic of China; 2 Sir Run Run Shaw Hospital, Zhejiang University, Hangzhou, 310018 People’s Republic of China; 3 The Second Affiliated Hospital of Zhejiang Chinese Medical University (The Xin Hua Hospital of Zhejiang Province), No. 318 Chaowang Road, Hangzhou, 310005 Zhejiang People’s Republic of China

**Keywords:** Intestinal microbiota, Hashimoto thyroiditis, Gut-thyroid axis, Biomarker, Autoimmune disease

## Abstract

Intestinal microbiota plays an indispensable role in the host's innate immune system, which may be related to the occurrence of many autoimmune diseases. Hashimoto thyroiditis (HT) is one of the most common autoimmune diseases, and there is plenty of evidence indicating that HT may be related to genetics and environmental triggers, but the specific mechanism has not been proven clearly. Significantly, the composition and abundance of intestinal microbiota in patients with HT have an obvious difference. This phenomenon led us to think about whether intestinal microbiota can affect the progress of HT through some mechanisms. By summarizing the potential mechanism of intestinal microflora in regulating Hashimoto thyroiditis, this article explores the possibility of improving HT by regulating intestinal microbiota and summarizes relevant biomarkers as therapeutic targets, which provide new ideas for the clinical diagnosis and treatment of Hashimoto thyroiditis.

## Introduction

Hashimoto thyroiditis (HT) is a kind of chronic autoimmune thyroiditis. According to statistics, the global prevalence of HT has reached 10–12%, with a high incidence of 30–50 years old and female to male predominance of 7–10:1 [1–3]. In the past decades, the incidence rate of HT has gradually increased, which has become a global public health problem [4, 5]. Furthermore, in areas of iodine sufficiency, HT is the most common cause of hypothyroidism in adults [6]. In a study on the incidence rate of thyroid diseases among people in regions with low, sufficient or excessive iodine intake in China, the cumulative incidence rate of HT was 0.2%, 1%, and 1.3%, respectively [7]. Pathologically, Hashimoto thyroiditis is characterized by lymphoplasmacytic infiltration, tissue fibrosis, lymphatic follicular formation, parenchymal atrophy and acidophilic change of follicular cells [1]. The most common clinical manifestation is goiter with or without hypothyroidism. The pressure of the enlarged thyroid on the neck can lead to dysphonia, dyspnea and dysphagia. Due to the loss of thyroid function and primary hypothyroidism, HT patients often have other symptoms involving multiple systems of the body. Gastrointestinal symptoms are always caused by changes in serum thyroid hormone, among which constipation is the most common clinical symptom. The intestinal peristalsis of HT patients is often significantly weakened because of decreased thyroid hormone, which even lead to pseudo-obstruction or intestinal obstruction. In addition, the hypotension of gallbladder and changes in bile composition caused by low thyroid hormone will lead to an increase in the formation of bile duct stones [8]. The autoimmune performance of HT is based on the interaction between environmental factors and genetic susceptibility [9], but its specific pathogenesis has not been clarified. Because HT usually accompanies patients for life and is difficult to recover, which often seriously affects the normal work and quality of life of patients. At present, there is still a lack of effective treatment methods, so it is urgent to find an effective intervention for curing disease.

There are trillions of microorganisms in the human gastrointestinal tract, forming a reciprocal relationship with the host. Intestinal microbiota plays a key role in maintaining the homeostasis of the digestive tract, limiting the colonization of pathogens, and regulating metabolism [10–12]. At the same time, the microbiota is the basis for the correct development of gut-associated lymphoid tissue (GALT) and initiates the immune response of GALT through pattern recognition receptor/pathogen-associated molecular patterns (PRR–PAMP) recognition and epigenetic regulator (such as short-chain fatty acid) [13]. In return, the host provides the living environment and nutrients for the gut microbiota and affects the composition of the microbiota through the immune response induced by the symbiotic flora [14, 15]. Therefore, the imbalance between the host immune system and intestinal microbiota may lead to disease. The research on intestinal microbiota mostly focuses on gastrointestinal diseases. In clinical practice, the treatment based on regulating intestinal microbiota has been applied to inflammatory bowel disease [16], irritable bowel syndrome [17], and liver cirrhosis [18], which has become a hot research topic in the world. However, changes in intestinal microbiota are also described in some extraintestinal diseases, such as type 2 diabetes [19], rheumatoid arthritis [20], systemic lupus erythematosus [21], and other autoimmune diseases.

In recent years, with the in-depth study of intestinal microbiota, the correlation between intestinal flora and HT began to receive attention. Both animal models and patients have confirmed that HT can affect the composition and abundance of intestinal microorganisms, leading to dysbiosis of intestinal microbiota [22–24]. On the one hand, the dysbiosis of intestinal microbiota leads to the damage of the gut barrier, and then bacterial translocation, which destroys the immune tolerance in thyroid autoimmunity through a series of mechanisms, including molecular simulation, bystander activation, and epitope spreading [12]. On the other hand, increasing evidence shows that microbiota regulates the immune response, changes the balance of T cell subsets, affects the absorption of trace elements and thyroid hormone metabolism through the gut-thyroid axis, and finally affects the thyroid function [25, 26]. In addition, the bacterial antigen may enhance the activation of inflammasomes (like NLRP1, NLRP3, NLRC4, and AIM2) and increase the expression of related cytokines by binding with antibodies in blood [27, 28]. In addition to the microbiota, its metabolites, such as short-chain fatty acids and secondary bile acids, also play an important role in the gut-thyroid axis [29]. In this article, we will discuss the above mechanisms, explore the possibility of improving HT patients by interfering with intestinal microbiota, and summarize the level changes of relevant biomarkers in HT patients to discuss the potential as a new diagnosis and treatment marker, such as zonulin protein, inflammatory body, short-chain fatty acid, secondary bile acid, IL-18, and IL-1. It is helpful to provide theoretical support for the further development and application of microecological therapy in HT.

## Interaction between gut microbiota and thyroid gland: gut-thyroid axis

### Dysbiosis in HT

The intestinal microbiota is mainly composed of bacteria, viruses, fungi, prokaryotic communities and other microorganisms, in which aerobic bacteria and anaerobic bacteria are the main bacteria. In the human healthy intestine, the most representative bacterial phyla are Firmicutes and Bacteroides, followed by Proteobacteria, Actinomyces, Fusobacteria and Verruciformes [12, 30, 31]. Under normal circumstances, the interaction between intestinal microbiota and host maintains a dynamic balance, however, when the human micro-ecosystem exceeds the self-regulation ability, intestinal dysbiosis can lead to the occurrence of disease [32, 33]. Studies in different regions have compared the differences in microbiota between HT patients and healthy people, and various data support the occurrence of intestinal dysbiosis in HT (Table 1). In a meta-analysis demonstrating the relationship between intestinal microbiota and autoimmune thyroid disease (AITD), researchers found that beneficial bacteria (such as *Lactobacillus* and *Bifidobacterium*) in the intestines of patients with AITD decreased and harmful microbiota (such as *Bacteroides fragilis*) increased significantly [34]. Zhao et al. observed that the composition of intestinal microbiota in HT patients were significantly different from the composition of intestinal microbiota in the healthy people: the relative abundance of *Blautia, Roseburia, Ruminococcus_torques_*group, *Romboutsia*, *Dorea*, *Fusicatenibacter* and *Eubacterium_ hallii_* group genera increased, while the relative abundance of *Fecalibacterium*, *Bacteroides*, *Prevotella_9* and *Lachnoclostridiuum* genera decreased [35]. Several other studies also confirmed the reduction of *Faecalibacterium*, *Bacteroides*, *Prevotellaceae* and *Lachnoclostridium* [36–38]. In addition, research that the proportion of *Firmicutes/Bacteroidetes* (F/B) in the intestinal microbiota of HT patients increased. Because Firmicutes and Bacteroidetes are the main dominant microbiota at the level of the phyla, their proportion is related to the disease susceptibility, which may reflect the ecosystem of the gastrointestinal tract to infer the disease status of HT [35, 39]. Ishaq et al. used 16S rRNA gene sequencing to analyze the microbiota in HT, the findings showed that at the gate level, the abundance of Proteobacteria and Cyanobacteria was higher, the level of Actinobacteria was improved, while the abundance of Firmicutes and Bacteroides was lower; at the level of families and genera, *Enterobacteriaceae*, *Alcaligenaceae* and *Parasutterella* genera increased, while *Prevotellaceae*, *Ruminococcaceae, Veillonellaceae* and *Dialister* decreased. At the same time, the data from real-time PCR showed that *Bifidobacterium* and *Lactobacillus* were significantly decreased in HT patients [36]. Interestingly, in another research, Liu et al. showed that the intestinal abundance of *Bifidobacterium* in HT patients increased with the development of the disease [37]. Moreover, some study results showed that the species richness index Chao1 of HT patients had a significant increase, which may indicate the excessive growth of intestinal bacteria [34, 36]. Although high microbial diversity is usually associated with better health outcomes, it also possibly causes destructive effects, such as increased protein breakdown and decreased polyphenol conversion, mucus secretion, and epithelial turnover [40]. Therefore, although the intestinal microbiota of HT patients has changed, whether it is to help the host fight against or promote disease needs more animal experiment to prove.

**Table 1:** Change of intestinal microbiota in HT patients

Cohort	Country	Method	Increase	Decrease	References
HT versus healthy individuals	Brazil	Real-time PCR	*Bacteroides*	*Bifidobacterium*	[24]
HT versus healthy individuals	China	16S rRNA gene sequencing	*Blautia* *Roseburia* *Ruminococcus* *Romboutsia* *Dorea* *Fusicatenibacter* *Eubacterium*	*Faecalibacterium* *Bacteroides* *Prevotella_9* *Lachnoclostridium*	[35]
HT versus healthy individuals	China	PCR-DGGE, Real-time PCR, 16S rRNA gene sequencing	*Proteobacteria* *Cyanobacteria* *Actinobacteria* *Enterobacteriaceae* *Alcaligenaceae*	*Firmicutes* *Bacteroidetes* *Veillonelliaceae* *Prevotellaceae* *Dialister* *Bifidobacterium* *Lactobacillus*	[36]
HT with ab/normal thyroid function versus healthy individuals	China	16S rRNA gene sequencing	*Akkermansia* *Lachnospiraceae* *Bifidobacterium* *Shuttleworthia* *Clostriworthdia*	*Lachnoclostridium* *Bilophila*	[37]
HT with ab/normal thyroid function versus healthy individuals	China	16S rRNA gene sequencing	*Phascolarctobacterium* *Lachnospiraceae_* *Lactonifactor* *Alistipes* *Subdoligranulum*	*–*	[41]
Primary hypothyroidism versus healthy individuals	China	16S rRNA gene sequencing	*Neisseria* *Rheinheimera*	*Veillonella* *Paraprevotella*	[23]
HT versus GD and healthy individuals	Spanish	16S rRNA gene sequencing	*Victivallaceae* *Streptococcus* *Rikenellaceae*	*Faecalibacterium*	[38]

### Effect of microbiota on the immune system

#### Microbiota and innate immunity

Commensal bacteria are recognized as the basis for the correct development of GALT. The innate immune cells in GALT, including innate lymphocytes (ILC), macrophages, dendritic cells, and intestinal epithelial cells (IECs), trigger the immune response by non-specific recognition of pathogens [42–44]. In healthy conditions, the intestinal microbiota forms a symbiotic relationship with the host and interacts with epithelial cells and immune mucosal cells to maintain immune homeostasis through PRR-PAMP recognition [15, 45, 46]. Intestinal microbiota plays a key role in regulating the development of antigen presenting cells (APCs) [47]. Atarashi et al. demonstrated that microbial-derived ATP can stimulate the dendritic cell subpopulation expressing CD70 and CX3CR1 on its surface, which induces the differentiation of Th17 cells [48]. Conventional natural killer (NK) cells, a kind of ILC, can produce IFN-γ, TNF-α and granulocyte–macrophage colony stimulating factor (GM-CSF) to participate in the immune response [49]. Sanos et al. found that GF mice lack NKp46 cells that produce IL-22, indicating that intestinal microflora may play a key role in promoting the differentiation of IL-22NKp46 cells [50]. In addition, intestinal microbiota was found to be dependent on the TLR-Myd88 pathway to promote the migration of mast cells to the intestine by inducing CXCR2 ligands from IECs [51, 52]. Professional antigen presenting cells (macrophages and dendritic cells) and ILC can block self-reactive cells and play a crucial role in autoimmune diseases [53, 54].

#### Microbiota and adaptive immunity

CD4+T cells are key components of the adaptive immune system. Previous studies have shown that the composition of microbiota affects the balance between the two main effector T cell populations (IL17+Th17 Treg and CD25+Foxp3+Treg) and induces the transformation of Th1 to Th2 [55–58]. A considerable number of microbial species have been confirmed to participate in intestinal immunity, for example, the segmented filamentous bacterium(SFB) in the intestine can induce Th17 cells in the intestinal lamina propria [59, 60]; Polysaccharide A (PSA) from Bacteroides fragilis can induce the expansion of CD4+T cells systemically and reverses the Th1 /Th2 imbalance, and exerts systemic anti-inflammatory activities by enhancing the production of IL-10 and by promoting the level and function of IL-10+Foxp3+Treg cells [61–63]. Based on the above findings, we have reason to assume that changes in the relative abundance of specific symbiotic microbiota in HT may affect the differentiation of effector T cells, thus affecting the process of disease. In addition, the components of microbiota may induce B cell activation factors and participate in the induction of IgA-producing B cells. Recently, it has been demonstrated that the immunogenicity of different bacterial strains plays a pivotal role in the induction of regulatory B cells, which are crucial in the suppression of inflammatory immune response. In different germ-free (GF) animal models (mouse, pig) [64, 65], it was observed that the number of macrophages and neutrophils decreased, CD4+ and CD8+ cells decreased, Th1 /Th2 regulation imbalance, Th17 and TREG differentiation decreased, IgA level decreased and plasma cell number decreased [47]. The above results show that intestinal microbiota plays an important role in regulating innate and adaptive immunity, and the absence of microbiota will cause serious immune system disorders.

#### Microbiota and thyroid autoimmunity

Microbiota can participate in the body's autoimmunity through a variety of mechanisms and play an important role in the development of autoantigen tolerance, including molecular simulation, bystander activation, and epitope spreading. Molecular simulation has been proven to be an extremely common mechanism. Through this mechanism, the microbiota can escape immune response and regulate the biosynthesis and metabolic pathway of the host [66–68]. The study found that some microbiota, such as *Lactobacillus*, *Bifidobacterium* and *Helicobacter pylori*, can induce thyroid autoimmunity by through molecular simulation, because some bacterial proteins have structural homology with human thyroid globulin (hTg)and thyroid peroxidase (TPO), and can selectively bind human TPO and Tg antibodies, and compete with natural antigens to bind autoantibodies [69, 70]. Bevenga et al. proved that 16 Borrelia proteins not only have significant amino acid sequence homology with thyroid stimulating hormone receptor (TSHR) but also with Tg, TPO, and sodium-iodide symporter (NIS) [71].

Secondly, sometimes the microbiota may not have antigens similar to the host structure, but it can induce co-stimulation and cytokine production through antigen presenting cells, leading to the activation of bystander T cells [72, 73]. Arata et al. showed that hTg-specific transferred cells had initiated bystander activation of naive host lymphocytes, which was confirmed to exist in HT [74]. They proposed that bystander activation might be achieved through the following ways: by provoking cell death and thus the release of cellular antigens, increasing the visibility or abundance of antigens; by attracting and potentiating antigen presenting cells; or by disturbing the cytokine balance through the inflammation that is associated with infection.

Finally, intramolecular epitope spreading is considered to be the mechanism of the expansion of autoimmune response to many sites of protein autoantigen; after the initial reaction to some dominant epitopes, antibodies against multiple secondary or recessive epitopes are produced by intramolecular epitope spreading [75, 76]. Thrasyvoulides et al. proved that peptide TgP41 not only includes the epitopes of Grave’s Disease (GD)-related autoantibodies, but also the main immunogenic epitopes of experimentally induced Tg specific antibodies, which can drive the spreading of B cell epitopes [76]. In addition, dysbiosis may produce new epitopes with auto-immunogenicity through post-translational modification of proteins, including citrullination and acetylation of active lysine, etc., and then induce autoimmune reactions [77]. In fact, in several autoimmune diseases such as rheumatoid arthritis (RA) and systemic lupus erythematosus (SLE), anti-citrulline protein antibodies have been used as good diagnostic markers in the clinic [78].

#### The intestinal barrier in HT

Under normal circumstances, the intestinal mucosal barrier can effectively prevent the pathogen from entering the circulatory system. However, when the integrity of the intestinal barrier is damaged, the submucosal immune cells are exposed to bacteria, dietary antigens and autoantigens, which can result in adverse immune activation or tolerance response failure, leading to autoimmune diseases [79]. Nowadays, there is a growing body of evidence proving that intestinal microbiota plays a crucial role in maintaining the integrity of the intestinal barrier [80]. In extreme cases, studies on germ-free animals confirmed that the lack of intestinal microbiota would lead to the degradation of intestinal mucosal barrier function, the specific manifestations are the reduction of the overall intestinal surface area, shorter intestinal villi, the reduction of intestinal crypt, the increase in intestinal permeability as well as the thinning and instability of mucous layer [81, 82]. A large number of studies show that intestinal dysbiosis, bacterial overgrowth and increased intestinal permeability (intestinal leakage) contribute to the development of HT [25, 40, 83]. Demir et al. found that the increased intestinal permeability caused by the change of intestinal microbiota was related to the higher level of zonulin, a protein responsible for regulating the intercellular connection [84]. Zonulin is a paracrine signal protein secreted by small intestinal epithelial cells, a physiological regulator of the tight connection between cells, and is involved in macromolecular transport, integrity of epithelial and endothelial barrier, and immune tolerance in intestinal mucosa [85]. Similar to the changes in patients with IBD, patients with HT also have abnormal intestinal barrier permeability. Therefore, it can be considered as one of the major causes of HT that the intestinal barrier function is damaged in the presence of inflammation, and the increased intestinal permeability leads to the antigen that could not pass through the intestinal barrier entering the systemic circulation and activating the immune system, and the generated antibody attacking the thyroid tissue [86].

#### Microbiota and autophagy defect

There is also interaction between intestinal microbiota and autophagy, which is closely related to the pathogenesis of thyroid autoimmune diseases. Autophagy is a highly conservative physiological process. The components in cells undergo lysosome-mediated self-digestion and circulation, and damaged or aged biological macromolecules and organelles are removed from the cytoplasm [87]. On the one hand, intestinal microbiota and its metabolites can control autophagy through mTOR pathway [88]. On the other hand, autophagy affects the composition of intestinal micro-organisms. It can be observed that the abundance of pro-inflammatory bacteria (such as *Candidatus arthromitus*) and pathogenic bacteria (such as *Pasteurellaceae* family) is increased, while the number of anti-inflammatory bacteria (such as *Akkermansia muciniphila* and members of the *Lachnospiraceae* family) is decreased in mice with intestinal specific autophagy-related gene 5 (ATG5)deficiency [89]. Meanwhile, autophagy defect will change the expression level of the tight junction protein claudin-2 in the intestinal mucosa. Different from the function of most tight junctions (such as occludin, claudin-1, ZO-1), the expression of claudin-2 protein increases the cell bypass permeability, leading to the destruction of the intestinal epithelial barrier and the increase in bacterial translocation and transmission, and then resulting intestinal dysbiosis [90, 91]. In addition to mediating inflammatory reaction and immunity, autophagy is also an important mechanism for clearing excess reactive oxygen species (ROS) to prevent cell damage and death. Autophagy defects lead to the accumulation of depolarized mitochondria and proteins and induce the release of inflammatory body activator (ROS or mitochondrial DNA) [92, 93]. As an important pathogenesis, inflammatory factors and ROS also participate in the inflammatory process of HT. Normally, ROS is essential for thyroid hormone synthesis, but excessive levels of oxidative stress can induce damage to thyroid follicular cells (TFCs) and promote the occurrence of HT by causing thyroid inflammation [94]. The elevated serum concentration of IL-23 can be detected in HT patients, which is because IL-23 promotes the development of HT by promoting Th17 cell differentiation and IL-17 secretion [95, 96]. Recent evidence has revealed a new mechanism that IL-23, as a strong inducer of AKT/mTOR signal pathway, can inhibit the autophagy activity ofTFCs, resulting in excessive ROS accumulation [97]. In addition, Lu et al. also found that Caveolin-1 deficiency inhibited the autophagic activity of TFCs and induced AKT /mTOR activation, which may be involved in the pathogenesis of HT [98]. In turn, excessive ROS promotes the production of inflammatory reactions and proinflammatory cytokines and IL-23. This forms a positive feedback cycle and aggravates the severity of the disease [99].

### Microbiota and metabolites regulate inflammation

The intestinal microbiota has been proven to be able to participate in the regulation of body inflammation. Microbiota can activate pro-inflammatory or anti-inflammatory processes, and the imbalance between the two will trigger the thyroid autoimmune process [32]. The increased intestinal permeability caused by dysbiosis makes the antigen enters the circulation and activates the immune system. At the same time, the antibodies in the blood may react with bacterial antigens and enhance the activation of inflammatory bodies in the thyroid [34]. Intestinal microbiota can increase the activation of inflammasome through flagellin, type III secretory system (T3SS), lipopolysaccharide (LPS), toxin, etc. Among them, the expression of NLRP3 mainly locates in the TFCs near the lymphoid infiltration area, which can recognize bacteria, viruses or other pathogens, and promote the activation of caspase-1 precursor to caspase-1, thereby activating IL-1 β and IL-18 [100]. IL-1β can promote the recruitment and activation of neutrophils and dendritic cells, and differentiation of Th17 and Tregs. At the same time, IL-1β induces the expression of Fas/FasL and intercellular adhesion molecule-1 on TFCs and interferes with the integrity of thyroid epithelium [101]. IL-18 can activate intestinal epithelial cells to produce antimicrobial peptides (AMPs) and stimulate the production of Th1, NK and NKT to secrete IFN-γ, or combine with IL-12 to induce more IL-17-secreting γδ T cells [102, 103]. The abnormal expression or dysfunction of the inflammasome is related to autoimmune and organ damage as well as various diseases and has been confirmed in autoimmune diseases, such as multiple sclerosis and lupus nephropathy [104, 105]. Guo et al. confirmed the relationship between HT and the expression level of inflammatory corpuscles. They found that the significantly enhanced expression of NLRP1, NLRP3, NLRC4, AIM2, ASC, caspase-1, pre-IL-1β, pre-IL-18, mRNA and protein in thyroid tissue of HT patients [28]. Therefore, they proposed the mechanism that inflammasome contributes to the development of HT, that is, the activation of inflammasome in TFCs induces immune response by mediating cell death and release of bioactive cytokines. It can be seen that intestinal microbiota induces the expression of some inflammatory cytokines and chemokines by enhancing the activation of inflammasome and then promotes the inflammatory reaction to participate in the pathogenesis of HT.

Lipopolysaccharides (LPS) are the main components of the cell wall of Gram negative bacteria, which can reflect the level. The binding of LPS to Toll-like receptor 4 (TLR-4) activates a wide range of cellular signaling pathways, such as NF-aκB and MAPK pathways, thereby inducing chronic subclinical inflammatory processes and TNF, IL-1 β Expression and secretion of pro-inflammatory cytokines such as IL-6. [106]. The latest research proposes the key mechanism of thyroid homeostasis disorder caused by bacterial LPS, including increased expression of Tg and NIS gene, TLR4-κB (NF-kB) pathway and regulating the activities of different deiodinase (type I and type II) [107].

In addition to the microbiota itself, various metabolites produced by the interaction between the host and the microbiota also participate in the regulation of inflammatory responses. Short-chain fatty acids (SCFAs) are metabolites secreted by intestinal microbiota during the fermentation of dietary fiber, mainly including butyrate, acetate and propionate (the ratio is 60:20:20), which can affect immune regulation and have anti-inflammatory effects [29]. It has shown that SCFAs can inhibit the inflammation that induced by LPS through G protein-coupled receptors (GPCRs) and histone deacetylases (HDACs). Free fatty acid receptor 3 (FFAR3) and FFAR2 are the most important GPCR receptors of SCFAs. SCFAs can inhibit IL-6, IL-1β and TNF-α by activating FFAR receptors to exert anti-inflammatory effects [108]. Butyrate is regard as the most effective inhibitor of HDACs, and it can reduce IFN-γ by inhibiting HDAC, while resulting in increase of naive CD4+T cells and Treg cells [109], and inhibiting TNF and the activity of NF-κB in monocytes and neutrophils activated by LPS [110, 111], and increasing the IL-10 produced by macrophages to regulate T cell activity in other tissues [112], and is related to the inhibition of NLRP3 inflammasome activation through GPR109A [113–115]. In the other hand, SCFAs can induce intestinal cell differentiation together with thyroid hormones, which enhances the integrity of the epithelial barrier, and participate in the regulation of the thyroid-gut axis [116].

Another important substance, bile acid (BA) metabolites, are produced by microbial biotransformation of BA that pass through the enterohepatic circulation, which plays an important role in regulating intestinal inflammation by regulating immune cell maturation and cytokine release through farnesoid X receptor (FXR) and G protein-coupled receptor 5 (TGR5) receptors [117, 118]. There are studies confirming that BA exerts anti-inflammatory effects by affecting the development of RORγ+Treg cells in the colon of mice [119]. In addition, trimethylamine N-oxide (TMAO), an intestinal microbiota-dependent product, was found to enhance the activation and formation of NLRP3 inflammasome, ASC, IL-1b and caspase-1 [120]. Interestingly, microbial metabolism also has a strong impact on cytokine production, which is mainly mediated by the tryptophan metabolite tryptophol that has strong inhibitory effects on the TNF-α response [121].

### Intestinal microbiota affects thyroid function

Trace elements have a prominent impact on the interaction between microbiota and thyroid and participate in the conversion and metabolism of thyroid hormones, thus affecting the function of the thyroid. It is well known that iodine is an essential element for the synthesis of thyroid hormone, and intestinal microbiota has an obvious regulatory effect on iodine, mainly including two aspects: One is to affect the concentration of thyroid hormone by controlling the intake, degradation and hepato-enteral circulation of iodine [24]; and the second is to regulate the expression and activity of NIS through LPS and metabolites to affect the iodine metabolism of the thyroid [122]. If long-term exposure to a high iodine environment may increase the immunogenicity of thyroglobulin and then activate the autoimmune reaction, leading to the destruction of thyroid tissue [123]. For microbiota, the adverse effects of high-dose iodine may be caused by the oxidation of cytoplasmic and membrane components [124]. Liu et al. found that excessive iodine promotes pyroptosis of thyroid follicular epithelial cells in HT through the ROS-NF-κB-NLRP3 pathway [125].

The thyroid is the largest selenium (Se) reservoir in the body. Selenium can promote the activity of CD4+CD25+FOXP3+regulatory T cells (Treg), inhibit the secretion of cytokines, prevent the apoptosis of follicular cells, and avoid the production of cytokines in thyroiditis [126]. Selenium deficiency will also lead to the reduction of hormone and enzyme activity and the decrease of peripheral T3 synthesis [127]. Calomme et al. found that *Lactobacillus* converts sodium selenite in cells into selenocysteine and selenomethionine, thus promoting the absorption of selenium as organic selenium by the human body, which proves that the metabolism of selenium is affected by microbiota [128]. In addition, intestinal microbiota can isolate selenium and limit the availability of the host [129]. The resident microbiota in the colon metabolize selenium, so it will not be absorbed by the upper digestive tract. In turn, selenium affects the composition and colonization of intestinal microbiota. Kasaikina found that selenium changed the diversity of microbiota in mouse models, which indicated that, *Porphyromonadaceae* phylotypes 1 and 3 and *Tannerella* phylotype 2 had increased reaction to selenium, while *Alistipes phylotype* 1 and *Parabacteroides* phylotype 3 declined [129].

Iron (Fe) and zinc (Zn) are also important elements supporting thyroid function. Iron is necessary for the effective use of iodine and the synthesis of thyroid hormone. Iron deficiency will lead to thyroid hormone impaired synthesis, storage, and secretion [130]. On the one hand, iron affects the composition of microorganisms. In the mice model, an iron-supplemented diet changed microbial diversity and increased the abundance of *Clostridiales* and *Lactobacillales* [131]. Because siderophores of bacteria (mainly *Enterobactin*) have a high affinity for iron, it provides a heme-rich condition for the growth of pathogenic bacteria [132]. On the other hand, by producing SCFAs to reduce the pH, the microbiota can improve iron bioavailability in the colon [133]. Iron supplementation increased *Enterobacteriaceae* and *Bacteroides* and decreased *Lactobacillus* and *Bifidobacterium*, with the decrease in butyrate and propionate [134, 135]. This change is explained by the role of inflammation in promoting the microbiota [40].

In addition, the microbiota can affect the level of zinc in the body, and the availability of zinc in turn determines the status of intestinal flora. In broiler chickens with chronic zinc deficiency, it was observed that the intestinal population of *Proteobacteria* was significantly increased, and the number of Firmicutes correspondingly decreased [136]. Zinc deficiency can affect the synthesis of thyrotropin releasing hormone (TRH), and the levels of thyrotropin (TSH), T3 and T4 will decrease [137]. In return, hypothyroidism can also lead to zinc deficiency.

At present, the role of microbiota in iodothyronine metabolism has been revealed. The microbiota can uncouple the sulfated glucuronide derivatives of iodothyronine via bacterial sulfate esterase or β-glucuronidase to improve the reabsorption of thyroid hormone in enterohepatic circulation [26, 138]. On the other hand, inhibition of 5-deiodinase activity by microbiota reduces the transformation of T4 to T3 and rT3 [139]. A study based on the rats model found that intestinal bacteria can absorb unbound iodothyronine and even compete with albumin to bind thyroid hormone [25, 139]. It is worth mentioning that some microbiota also plays a role in stabilizing thyroid hormone fluctuations and improving the utilization of levothyroxine. For example, Escherichia coli can act as a reservoir of T3 by combining T3 with bacterial thyroid-binding protein [40]; *Lactobacilli* and *Bifidobacteriaceae* have a significantly lower adjustment requirement of T4, indicating that the supplement of microbiota has increased levothyroxine availability and stabilizes thyroid function [140].

### Effect of thyroid on the gastrointestinal tract

**Fig. 1:**
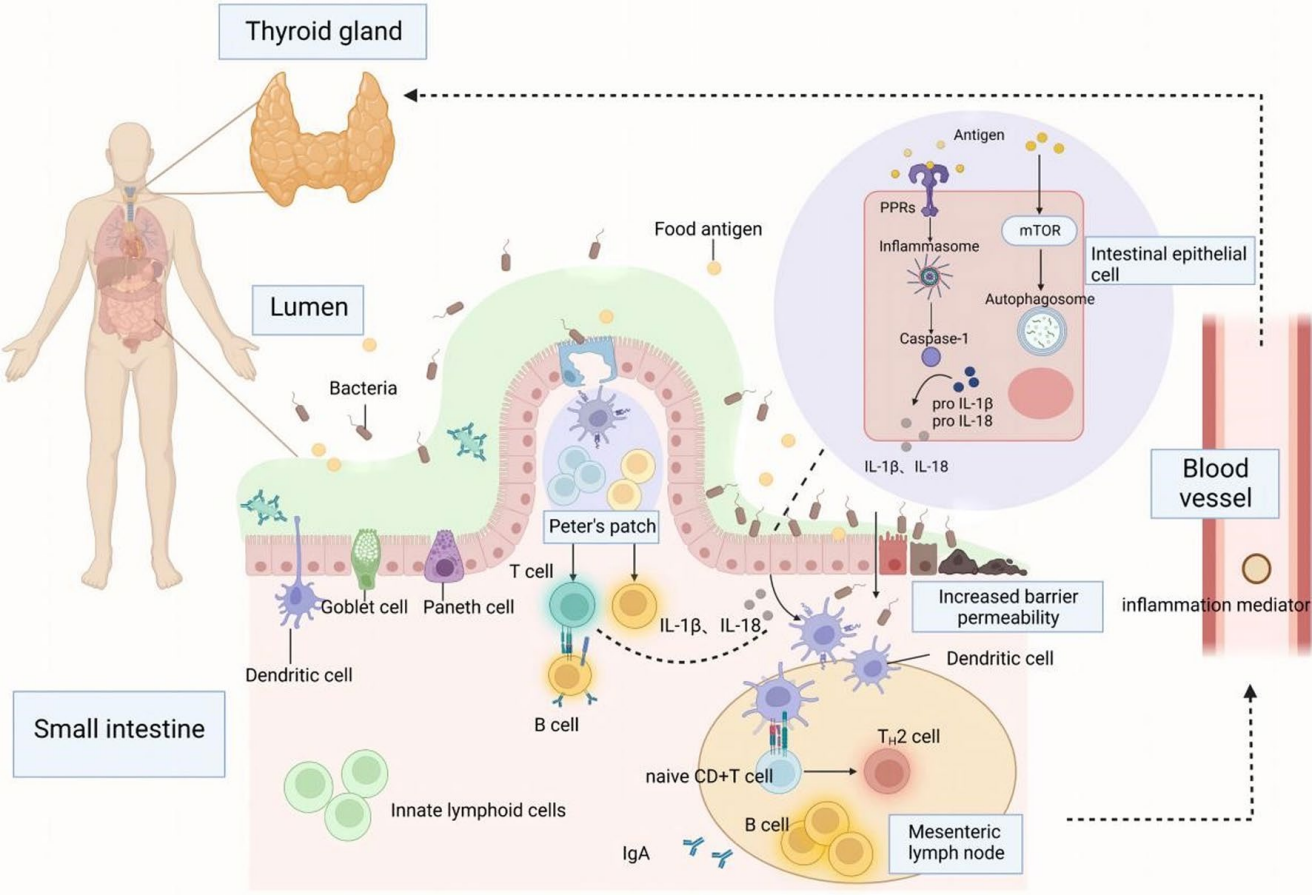
In steady-state conditions, microorganisms and their metabolites or food source antigen are the basis of intestinal-related lymphoid tissue (GALT) correct development, through pattern recognition receptor/pathogen-related molecular pattern (PRR–PAMP) recognition start intestinal immunity, promote the dendritic cells, T cells, B cells, innate lymphocyte differentiation or maturation. However, when the intestinal barrier integrity is destroyed and the intestinal mucosal permeability is increased, the intestinal flora and its metabolism can promote caspase-1 precursor activation to caspase-1 through the NLRP 3 inflammasome, and then activate IL-1 β and IL-18, and can also control autophagy through the mTOR pathway. When the intestinal inflammatory cascade expands and the inflammatory products enter the circulatory system, it may cause the occurrence and development of Hashimoto's thyroiditis

Previous studies have confirmed the role of thyroid function in the gastrointestinal system. T3 is considered to be the most important regulator of the development and differentiation of intestinal epithelial cells [12], and the lack of T3 is often an important reason for hypothyroidism. The variation in blood concentration of thyroid hormone causes a change in gastrointestinal neuromotor function, leading to the occurrence of gastrointestinal dysfunction, which can be clinically manifested as constipation and intestinal obstruction [141]. Diarrhea in hypothyroidism is mainly due to the increase in bacterial growth caused by intestinal hypomotility, and excessive bacteria contribute to damage to gastrointestinal neuromuscular function [142]. At the same time, severe hypothyroidism may lead to esophageal peristalsis. When the proximal end is involved, mucous edema causes difficulty in swallowing, while the distal end may have esophagitis and hiatal hernia [141]. A gastric myoelectric study by Gunsar et al. showed a positive correlation between dyspepsia and hypothyroidism score [143]. Due to muscle edema and decreased myoelectric rhythm, patients with hypothyroidism often have gastric motility disorders, resulting in delayed gastric emptying and gastric acid deficiency. In particular cases, hypothyroidism may also be responsible for refractory gastrointestinal bleeding in routine treatment [144]. On the other hand, thyroid function will also affect intestinal microbiota. Studies by Lauritano et al. showed that patients with significantly decreased thyroid function are more likely to have intestinal bacterial overgrowth [142] (Fig. 1).

## The relationship between HT and related digestive diseases

The relationship between gastrointestinal tract and thyroid was proposed as early as the 1950s and was called "thyrogastric syndrome." This consistency can be explained by the origin of the common germ layer and the specific composition of the microbiota. In recent years, the common mechanism between Hashimoto's thyroiditis and digestive tract diseases has attracted attention [40].

### HT and celiac disease (CD)

Celiac disease is a small intestinal inflammatory disease with autoimmune feature, which is triggered and maintained by the ingestion of the storage proteins (gluten) of wheat, barley, and rye. Because the molecular structure of glutenin is similar to that of thyroid tissue, it can lead to multiple autoimmune thyroid diseases. A single-center retrospective case–control study by Bibb ò et al. showed that HT is the most common autoimmune disease in patients with celiac disease [145]. In addition, their symptoms often overlap, so we have reason to believe that they may be connected through the gut-thyroid axis. Non-celiac wheat sensitivity (NCWS) is a non-allergic and non-autoimmune disease. It can also increase the expression and activation of TNF-α through toll-like receptors (such as TLR2 and TLR4) [146]. According to the gut-thyroid axis in HT, the pathogenesis of CD and NCWS can be explained from the following aspects: shared cytokine, molecular simulation, abnormal post-translational modification of proteins, malabsorption of essential micronutrients for the thyroid and damaged intestinal barrier [25].

### HT and Hp infection gastritis

In recent years, massive studies have already demonstrated that *Helicobacter pylori* (Hp) infection is closely related to the incidence of peptic ulcer and gastric cancer. During the past 2 decades, the correlation between Hp and other non-gastrointestinal diseases has been revealed. The relationship between thyroid autoimmunity and Hp can be explained by molecular simulation. Cellini M et al. described three mechanisms that ultimately activate Th1 autoreactive lymphocytes to link Hp and HT. Firstly, CD4+T cells recognize Hp epitopes with a similar structure to H/K/ATPase on the thyroid and activate Th1 to induce apoptosis. Secondly, dendritic cells present Hp epitopes to naive T cells, with a lack of peripheral immune tolerance, the Th1 will be activated. Finally, INF-γ can stimulate MHCII expression in follicular thyroid cells [147, 148]. In addition, Hp can produce a cytotoxin-associated gene A (CagA). It has been found that the cag-A positive Hp strains show a nucleotide sequence similar to the thyroid peroxidase (TPO) sequence, illustrating that serum CagA positive increases the risk of autoimmune thyroid disease [149].

### HT and autoimmune atrophic gastritis(AAG)

HT is found in nearly 40% of patients with autoimmune atrophic gastritis (AAG), who have a large number of anti-parietal cell antibodies in the serum [150]. Because gastric acid secretion is greatly reduced in AAG, iron absorption is too poor to promote the synthesis of T3 and T4. In addition, it is found that the levels of serum gastrin, chromogranin A and the proliferation of enterochromaffin-like cells (ECL) were significantly related to the coexistence of autoimmune diseases [151].

The above findings provide theoretical support from multiple perspectives. Therefore, we can further understand the role and mechanism of gut-thyroid by exploring the relationship between HT and other gastrointestinal diseases, and then broaden the clinical thoughts of HT and its complications, develop new therapies and drugs, and finally further improve the diagnosis and treatment level of HT.

## Potential therapeutic strategies by regulating the gut-thyroid axis

Although L-thyroxine (LT4) is currently a common treatment option for HT, it can only alleviate the lack of thyroxine, but cannot completely cure HT. Therefore, it is necessary to find an effective treatment by exploring the mechanism. To sum up, intestinal microbiota can effectively regulate the process of HT by increasing intestinal permeability, molecular simulation, bystander activation, epitope spreading, activation of inflammatory bodies, autophagy defects, trace elements absorption, thyroid hormone metabolism, and so on. At the same time, the classification and metabolic pathway level of intestinal microbiota reflect the interaction between intestinal microbiota and thyroid hormone, which allows us to give more consideration to traditional drug treatment for HT patients. Therefore, based on the relevant mechanism of the gut-thyroid axis, we summarized the following treatment strategies as auxiliary interventions.

### Intestinal non-absorbable antibiotics

For small intestinal bacterial overgrowth (SIBO) in HT patients, the treatment goal is to alleviate symptoms by eradicating bacterial overgrowth [152]. Antibiotic treatment is the basis for treating SIBO. Appropriate antibiotic treatment can improve the symptoms of gastrointestinal neuromuscular function damage by reducing the gastrointestinal bacterial burden, and then changing the composition of microbiota to regulate intestinal permeability. A meta-analysis of 10 prospective clinical studies on the treatment of SIBO patients with non-systemic antibiotics showed that the normalization rate of breath test in the antibiotic group was higher than that in the placebo group, indicating the effectiveness of antibiotics in regulating the flora [153]. Rifaximin is the first non-aminoglycoside intestinal antibiotic, which acts locally in the intestine and is not absorbable after oral administration, and is characterized by a broad antibacterial spectrum and strong antibacterial activity. The research results of Lauritano et al. showed that the overall incidence of adverse events of rifaximin was significantly lower than that of metronidazole group, showing that the side effects of non-absorbable antibiotics were less [154]. The results of a drug resistance study showed that the concentration of stool Staphylococcus spp and stool Coliforms would be temporarily reduced during the treatment of rifaximin [155]. Xu et al. showed that rifaximin changed the bacterial population in the ileum of rats, increasing the relative abundance of Lactobacillus, and preventing mucosal inflammation and impaired intestinal barrier [156]. Maccaferri et al. found that rifaximin did not affect the overall composition of intestinal microflora, but caused an increased concentration of Bifidobacterium, Atopobium and Faecalibacterium prausnitzii, accompanied by increased short-chain fatty acids [157]. There is evidence to support that a higher dose of rifaximin (1200–1600 mg/d) is an effective treatment method, which can achieve SIBO purification without increasing the incidence of side effects [158, 159].

### Rebuild intestinal ecology

Because antibiotics can only remove bacteria but cannot restore normal flora, antibiotic treatment alone cannot completely solve the microbial ecological imbalance related to SIBO. Therefore, it is necessary to think about how to rebuild intestinal flora, and the application of probiotics has become an attractive choice. Generally, we define some microbiota that is beneficial to the stability of microbiota as probiotics, which can enhance the intestinal barrier function, reduce inflammatory reaction and stabilize the intestinal microbiota. Common probiotics include *Lactobacillus*, *Bifidobacterium*, *Lactococcus*, *Streptococcus* and *Enterococcus*. As mentioned above, *Lactobacillus* and *Bifidobacterium* can participate in the occurrence of HT through molecular simulation. However, *Lactobacillus* and *Bifidobacterium*, as probiotics, regulate the immune response, showing anti-inflammatory effects and protecting the body from pathogens [34]. Studies have shown that *Lactobacillus* protects TH17 cells and supports barrier integrity by secreting IL-22 and IL-17. A study on *Lactobacillus* reuteri found that the beneficial effect on the mouse thyroid was achieved by promoting the production of IL-10 and the enhancement of Treg cells [12]. In addition, in the context of intestinal barrier dysfunction and inflammation, the yeast *S. boulardii* has also been widely studied. Its beneficial effects are mainly through antibacterial and antitoxic activities and nutritional effects on intestinal mucosa [160]. In recent years, the role of *Akkermansia muciniphila* on the intestinal barrier has also been revealed, which improves the function of intestinal barrier by restoring the thickness of mucous layer and tight junction protein as well as producing specific antibacterial and bioactive lipids with anti-inflammatory properties [161]. A study on the treatment of Grave's disease with probiotics supplemented with methimazole showed that *Bifidobacterium longum* supplemented with methimazole could improve thyroid function and reduce the concentration of TRAb in patients with GD [162]. In another double-blind randomized placebo-controlled trial, the probiotic compound LAB4 showed an obvious immunomodulatory effect [163]. It can be seen from this that it is feasible to improve HT through probiotics controlling microbiota in future. In addition to its effect on the intestinal barrier and immunity, probiotics can also reduce the fluctuation of thyroid hormone. Spaggiari et al. found that compared with the control group, the regulatory demand of T4 in the experimental group was significantly reduced, manifesting that the mixture of *Lactobacillus* and *Bifidobacterium* (VSL#3) increased the utilization of levothyroxine and stabilized thyroid function. However, the results of this study also show that the intake of probiotics of this genus will not change the susceptibility of HT patients or improve hypothyroidism [140].

Prebiotics are a kind of non-digestible food ingredient that can improve host health by selectively stimulating the growth and/or activity of intestinal beneficial bacteria [164]. The carbohydrate hydrolase of the microbiota promotes the fermentation of prebiotics to produce hydrogen, methane, carbon dioxide, SCFA, and other products. Currently available prebiotics include human milk oligosaccharides (HMOs), lactulose and inulin derivatives. Talebi et al. showed that supplementation of synbiotics (a combination of probiotics and prebiotics) can relieve constipation in patients with hypothyroidism and is beneficial to thyroid function, but no effect on thyroid peroxidase has been observed [165]. Based on the fact that the beneficial effects of microbiota are mediated by the secretion of various metabolites, the concept of postbiotics is proposed. According to Tsilingiri et al., postbiotics include any substances released by or produced by the metabolic activities of microorganisms, which directly or indirectly have beneficial effects on the host [166]. At present, the available postbiotic drugs include cell-free supernatant, exopolysaccharides, enzymes, cell wall fragments, short-chain fatty acids, bacterial lysates, vitamins, phenol-derived metabolites and aromatic amino acids. In the existing studies, epigenetic elements show the characteristics of immune regulation, anti-inflammation, anti-oxidation and anti-cancer [167]. These potential mechanisms suggest possible strategies for epigenetic elements to regulate the flora of HT patients. Although probiotics, prebiotics and postbiotics can play a certain role in the prevention and treatment of autoimmune diseases, considering that most probiotics research depends on animal models, the exact relationship between probiotics and HT still needs to be further explored to provide a new way for clinical regulation of intestinal dysbiosis or thyroid function in HT patients.

Fecal microflora transplantation (FMT) is direct evidence of the interaction between intestinal microflora and various diseases. At present, the specific mechanism of FMT is not clear, but it can be speculated that the potential mechanism is the improvement of microbial diversity, donor phage regulation of the recipient flora and altered microbial metabolite production [168]. In patients with recurrent Clostridium difficile infection, FMT has achieved amazing results [169]. In 2020, China released the Chinese experts consensus on standardized methodology and clinical application of fecal microbiota transplantation, and FMT was included in the guidelines as treatment of recurrent or refractory Clostridium difficile infection [169]. Furthermore, this consensus pointed out that FMT would be gradually applied to the treatment of autoimmune diseases. In future, the research on the prevention and treatment of HT by FMT may have important clinical significance.

Significantly, due to individual differences, the different sensitivity of intestinal microbiota to biological intervention may lead to inconsistency in results. Therefore, the key to the individualized treatment of ecological therapy in future is to explore the premise of effective probiotics implantation and understand the degree of inter-individual variation of the sensitivity of microbiota to probiotics intervention.

### Dietary therapy and trace element intake

The regulation of diet on microbiota directly affects the characteristics of inflammation. Because of the rapid and repeatable response of microbiota to dietary intervention, the rational design of personalized diet has become an important microbiota regulation method [32]. A study of mice showed that after the transformation from a low-fat and high-fiber diet to a "Western-style diet" (WDs) with high sugar, high fat and low fiber, they had serious ecological disorders, resulting in loss of secretory IgA function, inhibition of Treg cells producing IL-10, damage of intestinal barrier, and immune imbalance [170]. In addition, mice on a Western diet showed increased Firmicutes phylum and decreased Bacteroidetes phylum. Similarly, the regulation of diet on microbial ecology has also been verified in human model research [171]. Different food types affect the production of microbial metabolism. Fiber, polyphenols, tryptophan and glucosinolates (vegetarian) increase SCFA, which is beneficial to Bifidobacterium population, while carnitine, choline and fat (a diet rich in animal protein) increase the production of secondary bile acids [40].

In addition, metabonomics research and germ-free mouse experiments showed that intestinal microbiota played a key role in the production of TMAO [100]. Symbiotic bacteria metabolize dietary lipid phosphatidylcholine and red meat component L-carnitine, which leads to the accumulation of TMAO, suggesting that our targeted inhibition of this reaction can reverse the accumulation of TMAO and improve the development of disease [172, 173]. The above results show that dietary fiber plays a crucial role in improving intestinal ecology. During the fermentation of dietary fiber, intestinal microbiota can produce SCFAs or metabolites with anti-inflammatory properties and maintain intestinal homeostasis. Therefore, it is suggested that the diet structure of HT patients should be adjusted accordingly: (1) Autoimmune Protocol (AIP) diet. Its basic principle is to avoid foods, additives, or medications (e.g., nonsteroidal anti-inflammatory drugs) that can trigger intestinal inflammation, dysbiosis, and/or symptomatic food intolerance. And it emphasizes eating more fresh and nutritious foods, bone soup and fermented foods, including a 6-week elimination stage and a 5-week maintenance stage [174]. A pilot study showed that AIP diet and lifestyle plan can reduce inflammation and regulate the immune system, significantly improving the health-related quality of life and symptom in middle-aged female HT patients [175]. (2) Anti-inflammatory diet. Focus on foods with high nutrition, such as whole grains with high fiber, vegetables rich in polyphenols and foods rich in omega-3 fatty acids. According to the current research results, the antioxidant properties of the Mediterranean diet may be the most beneficial for HT patients [176]. (3) A gluten-free diet. The study found that a gluten-free diet can improve the symptoms of malabsorption and hypothyroidism, reduce the demand for levothyroxine, reduce intestinal inflammation, and have a positive impact on the development and performance of autoimmunity [177, 178]. However, this diet is very strict and difficult to follow, which leads to the risk of nutritional deficiency. The current research has not confirmed that patients with HT should adopt a gluten-free diet, so it is not recommended [179].

Because iodine, selenium, zinc, vitamin D, etc., play a part in thyroid function through different ways of the gut-thyroid axis, excessive iodine intake, selenium deficiency and the use of specific drugs (such as amiodarone) are closely related to the onset of HT.

When probiotics are combined with different trace elements, they may have a synergistic effect on the host. Lactobacillus and Bifidobacterium seem to have a negative correlation with dietary iron, while a positive correlation with selenium and zinc [25]. In HT, the above two kinds of bacteria decrease, suggesting that intestinal flora may affect the intake of necessary trace elements for the synthesis of thyroid hormone. Therefore, the intake of trace elements can be reasonably adjusted according to the change of microbiota. For example, selenium supplementation can stimulate the immune system, inhibit the expression of human leucocyte antigen-DR (HLA-DR) in thyroid cells and reduce the thyroid autoimmune function [123]. In addition, vitamin D deficiency is one of the reasons for HT, and the more vitamin D deficiency, the greater the possibility of HT. Thomas et al. found that the diversity of microbiota is closely related to active vitamin D [180]. Studies have shown that 1α-25(OH)2D3, the active form of vitamin D, can balance the redox system and regulate immune tolerance, so vitamin D supplementation may alleviate the disease activity of HT. A double-blind randomized controlled trial found that the levels of TGAb and TSH in the vitamin D group were significantly lower than those in the placebo group, but the levels of TPOAb were not significantly lower [181]. Considering the existing research and the low cost and minimal side effects of vitamin D, it may be recommended to monitor and supplement patients with HT.

## Biomarkers provide new targets

Clinically, serum T3, T4, TSH and anti-thyroid antibody are common biological indicators for diagnosing HT. Recently, with the in-depth study of the mechanism of the gut-thyroid axis, more and more new biomarkers have been found. We summarized some important molecules mentioned above, and hope to provide new therapeutic targets for improving Hashimoto's thyroiditis by detecting these molecules (Table 2).

**Table 2:** Relevant biomarkers based on gut-thyroid axis

	Source	Mechanism	Significance	References
Zonulin/I-FABP/DAO	IECs	Adjust TJ	Reflect intestinal permeability	[84, 183] [184]
Claudin-2	IECs	Regulate cell bypass permeability	Increased claudin-2 expression is associated with autophagy inhibition	[90, 91]
NLRP1/ASC	TFCs	Mediates pyroptosis and cytokine (IL-1 β, IL-18) release to induce immune response	The mRNA level is correlated with the serum TPOAb and TgAb levels	[28]
NLRP3	TFCs	Mediates pyroptosis and cytokine (IL-1 β, IL-18) release to induce immune response	Increased expression in HT patients; Maintain intestinal microflora homeostasis	[188, 189]
TMAO	Microbial metabolites	Enhance the activation and formation of NLRP3, ASC, IL-1b and caspase-1	Promote inflammatory reaction	[111]
SCFAs	Microbial metabolites	Inhibit HDAC; Reduce INF-γ; Regulate T cell polarization; Strengthen TJs with thyroid hormone	Affect immune regulation, Anti-inflammatory Maintain intestinal barrier	[189, 190]
DCA	Microbial metabolites	Reduce bacterial overgrowth by inducing membrane damage	Signs of overgrowth of small intestinal bacteria	[194, 195]
LPS	Microbial metabolites	Increasing the expression of Tg and NIS genes; TLR-4 mediates the downstream activation of NF-kB; Affects the activity of deiodinase	Sensitive index of bacterial translocation; Cause thyroid homeostasis disorder	[122, 191–193]
H3K4me3	Histone	Abnormal PTMP	Epigenetic marker	[197, 198]
Cag-A	Helicobacter pylori	The nucleotide sequence of Cag-A positive Helicobacter pylori is similar to TPO sequence	Serum positive rate is related to AITD	[149]

The increase in plasma zonulin levels in HT patients suggests that zonulin may play a role in the pathogenesis of HT as a marker of intestinal permeability damage. Zonulin is the only physiological medium known to reversibly regulate intestinal permeability (IP) [182]. Under normal conditions, zonula occluden 1 (ZO-1) forms tight junction (TJs) complexes with the help of actin filament binding to form selective permeability of intestinal barrier [84]. In the presence of zonulin, activated epidermal growth factor receptor (EGFR) and protease-activated receptor 2 (PAR2) trigger the activation of phospholipase C, and then, protease-activated receptor α (PKC-α) is activated. Then the activated PKC-α triggers the release of ZO-1 and actin filament, which results in the loose conformation of TJs, increased barrier permeability and excessive abnormal antigenic transportation [84, 183]. In addition, intestinal fatty acid binding protein (I-FABP) and diamine oxidase (DAO) were also used to evaluate the integrity of the intestinal barrier and were verified. They are cytoplasmic proteins in intestinal epithelial cells, which are released into the circulation system immediately when the intestinal epithelium is destroyed. A study on Grave's disease pointed out that the increased levels of I-FABP and zonulin can damage intestinal epithelial cells through direct contact, toxin release and innate immune activation, which is probably due to the imbalance of microbiota. Although this study did not find the level difference of DAO, it inspired us to the biomarkers related to intestinal leakage [184].

Tschopp et al. first proposed the concept and composition of inflammasomes: (1) pattern-recognition receptors (PRR) or NOD-like receptors (NLR) or AIM2-like receptors (ALR); (2) apoptosis-associated speck-like proteins containing caspase recruitment domains (ASCs); and (3) caspase proteases [185]. As mentioned above, TFCs express toll-like receptors, which induce the activation of the innate immune system by responding to the PAMPs and damage-associated molecular patterns (DAMPs) on the surface of microorganisms, making lymphocytes product multiple proinflammatory cytokines, such as IL-1β, IL-18, IFN-γ, and TNF-α [186]. The pro-inflammatory cytokines secreted by infiltrating lymphocytes lead to an increase in the expression of inflammasomes in thyroid cells and enhance the activation of inflammasomes induced by DAMP [187]. The two mechanisms form a feedback loop to prolong the immune response of thyroid tissue. As one of the most important members of the inflammasome family, NLRP3 widely exists in epithelial cells and immune cells. Many microorganisms and their metabolites can regulate the expression of NLRP3. For example, Bacillus fragilis can activate the expression of NLRP3, and Hp can promote the activation of NLRP3 and caspase-1 and the secretion of IL-1b through TLR4, MyD88 and NF-kB [188]. It is worth noting that the subsequent effector molecule IL-1b downstream of the inflammasomes can regulate the microbiota by regulating the production of AMPs [189]. Guo et al. found that the mRNA levels of NLRP1 and ASC in thyroid tissue of AITD group were correlated with the levels of TPOAb and TgAb in serum, indicating that NLRP1/ASC may be a potential biomarker of AITD [28].

SCFAs are metabolites of dietary fiber fermented by symbiotic bacteria, and play a key role in the development, function and regulation of the immune system. SCFA, especially butyrate, can inhibit the production of pro-inflammatory cytokines induced by NF-кB in myeloid cells and promotes the production of regulatory T cells by inhibiting pro-inflammatory HDAC [189, 190]. Butyrate and propionate are HDAC inhibitors that epigenetically regulate gene expression. In addition to immunomodulatory and anti-inflammatory effects, butyrate is also the main energy source of intestinal epithelial cells, which can induce mucus production and strengthen the tight connection between cells together with thyroid hormone, playing an important role in maintaining the intestinal barrier [40]. Small-scale studies comparing HT and healthy human individuals found that the number of bacteria producing SCFA in HT decreased [35, 36], while the fecal and plasma levels of SCFA remained to be studied. Anaerobic Bacteroides, Bifidobacteria, Eubacteria, Streptococcus and lactobacillus are the main bacteria that produce SCFAs. Although the change in microbiota is diverse in species, it is more consistent in function. Therefore, as a product related to bacterial fermentation pathways, SCFA may be better used for disease monitoring and biomarker discovery.

Lipopolysaccharide (LPS) and D-lactic acid can be used as sensitive indicators of the translocation of intestinal bacteria and their metabolites. High levels of LPS and D-lactic acid indicate increased translocation. The effect of LSP on thyroid homeostasis helps to explain the pathogenesis of HT: LPS can directly affect thyroid cells by increasing the expression of thyroglobulin (Tg) and NIS genes [122]; secondly, LPS regulates thyroid cell function through TLR4-NF-kB pathway on thyroid cells [191]. In addition, as an endotoxin, LPS can inhibit the activity of hepatic type I iodothyronine deiodinase (D1) and conversely activates type II iodothyronine deiodinase (D2) in the hypothalamus and anterior pituitary, thus promoting the conversion of thyroxine (T4) to triiodothyronine (T3) [192, 193]. In addition, there is evidence that deoxycholic acid (DCA) may also become a marker of the gut-thyroid axis in HT. Primary bile acids (BAs) derived by Cholesterol are regulated by the nuclear FXR and the TGR5 and convert into secondary bile acids through 7α-dehydroxylation reaction, which includes deoxycholic acid and lithocholic acid [194, 195]. Many dominant genera in human intestines can produce secondary bile acids, of which Clostridium is the most active. Deoxycholic acid is believed to decrease bacterial overgrowth by inducing membrane damage [29]. According to the metabolic spectrum, the main bile acid of HT patients is DCA, which prompts the possible relationship between DCA and the overgrowth of intestinal bacteria in HT patients [196].

Microbial post-translation modification of protein (PTMP) is of great significance in inducing autoimmune diseases. Dysbiosis may contribute to autoimmune diseases through inappropriate PTMP. The modifications in the intestine include de/phosphorylation, de/acetylation, ubiquitination, methylation, citrullination, carbamylation, etc. Their enzymatic apparatus is capable to transform naïve/self or non-self-peptides to autoimmunogenic ones [77]. Based on an epigenetics study, Lu et al. showed that the increased protein levels of the trimethylation of histone H3 lysine 4 (H3K4me3) and Mixed lineage leukemia 1 (MLL1) in HT patients, which can be explained by the abnormal H3K4 trimethylation in lymphocytes and monocytes of thyroid tissue [197]. In addition, another study found that LPS stimulation could enhance H3K4me3 in macrophages [198]. CagA is a marker for the cag pathogenicity island, including the genes necessary for inflammation enhancement caused by pathogenic strains. A meta-analysis showed that the overall positive rate of cagA in patients with AITD was significantly higher than that in the control group, with significant differences [149]. Because the cag-A positive Hp strain showed a nucleotide sequence similar to the TPO sequence, there may be a cross-reaction between the antibody produced by Hp infection and the thyroid antigen, showing the significant correlation between cagA positive and AITD [147, 199].

## Discussion

Intestinal microbiota and thyroid interact with each other through the gut-thyroid axis and play a key role in the pathogenesis of HT. The excessive growth of small intestinal bacteria leads to dysbiosis, leading to intestinal barrier damage, bacterial antigen and its metabolites migrating to the outside of the intestine, which can increase the activation of inflammatory bodies and regulate inflammatory response. Cross-reaction, autophagy defect and functional imbalance of T lymphocyte subsets caused by intestinal dysbiosis directly lead to thyroid tissue damage. Trace elements affect the metabolism of thyroid hormone to participate in the development of HT through the role of intestinal microbiota. Although HT is closely related to intestinal dysbiosis, because of the numerous types and individual differences of intestinal microflora, it is necessary to deeply understand the structure and mechanism of intestinal microflora to provide more possibilities for accurate medical treatment of HT. In future, the treatment strategy of HT will be around the gut-thyroid axis gradually, aiming to slow down the progress of HT and improve the symptoms of patients by rebuilding the intestinal microbial ecology. Biomarkers can not only provide the basis for diagnosis but also guide the selection of intervention strategies. The occurrence of intestinal leakage suggests that we can intervene in the early stage of the disease, to restore the intestinal tight junction, repair the intestinal barrier, and prevent the intestinal immune cells and inflammatory factors from migrating out of the intestine. The restoration of intestinal homeostasis can select different antibiotics according to the changes in the microbial composition of HT patients and combine probiotics and other biological agents to eliminate pathogenic bacteria while restoring normal flora, which maintains an ingenious balance. In addition, the restoration of intestinal microbial ecology can be achieved through dietary changes, such as the intake of high-fiber and low-fat foods, which will promote the transformation of microbial metabolism to anti-inflammatory. However, since most studies on microbiota are based on animal models, it is necessary to make full use of clinical big data and more in vivo studies to verify the effectiveness of the above treatment schemes in future.

## Data Availability

Not applicable.
